# Predicting suitable habitats and conservation areas for *Suaeda salsa* using MaxEnt and Marxan models

**DOI:** 10.1016/j.isci.2025.112933

**Published:** 2025-06-18

**Authors:** Yongji Wang, Zhusong Liu, Kefan Wu, Jiamin Peng, Yanyue Mao, Guanghua Zhao, Fenguo Zhang

**Affiliations:** 1 School of Life Science, Shanxi Engineering Research Center of Microbial Application Technologies, Shanxi Normal University, Taiyuan, Shanxi 030000, China; 2 School of Life Science, South China Normal University, Guangzhou 510631, China

**Keywords:** Global change, Plant Biology, Plant biogeography

## Abstract

*Suaeda salsa* (L.) Pall. (*S. salsa*) is a salt-tolerant plant with medicinal and ecological value. Understanding its potential distribution under changing climate conditions is essential for conservation and sustainable use. Using 130 occurrence records and 14 selected environmental variables, this study applied the MaxEnt model to predict suitable habitats of *S. salsa* across China under current and future climate scenarios. The Marxan model was used to identify priority conservation areas based on habitat suitability and planning cost. Results show that temperature-related variables, soil salinity, and silt content are the most influential factors. Future scenarios suggest an overall expansion of suitable areas and a southeastward shift in habitat centroids. Priority conservation zones are primarily located in northern China, aligning with highly suitable habitats. This research provides a spatial basis for ecological restoration, habitat management, and future planning for *S. salsa* conservation under climate change.

## Highlights

• MaxEnt model predicts suitable habitats of *Suaeda salsa* under climate change scenarios
• Marxan model identifies priority conservation areas based on habitat suitability
• Temperature and soil salinity are key environmental factors influencing distribution
• Future suitable habitats expand and shift southeastward under high-emission scenarios

## Graphical abstract

**Figure:**
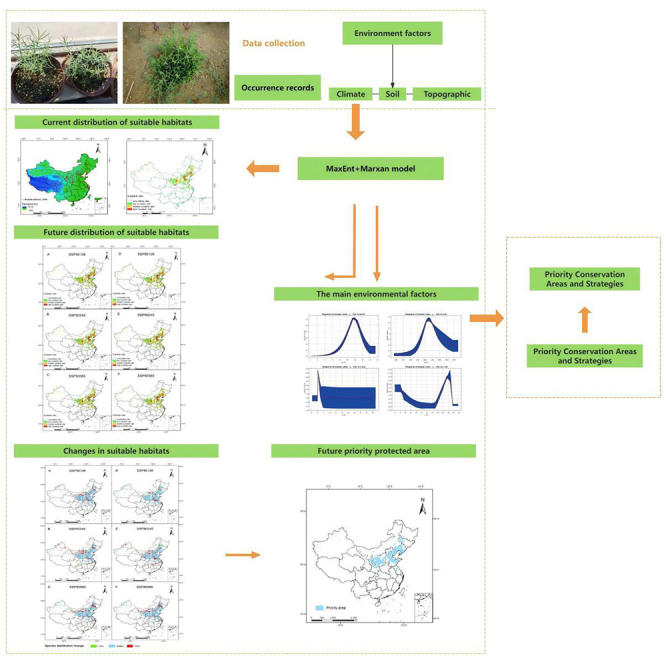

## Introduction

Global climate change continues to impact the growth and development, geographical distribution, and population size of plants. Global warming has profound effects on plant communities.^1^ Research indicates that climate change could lead to habitat destruction and become the biggest threat to global biodiversity in the coming decades.^2^ With the occurrence of global warming progresses, some species will migrate to higher latitudes or altitudes, whereas others may adapt to these changes physiologically or phenologically, These changes present significant challenges for the artificial cultivation and cultivation of medicinal plants, and ongoing climate change has the potential to lead to the extinction of many medicinal plants.^3^ This situation poses a serious threat to the continued use of medicinal plants. Therefore, studying the response mechanisms of plants to climate change has important reference significance for future climate adaptation and plant protection.

Understanding the effects of climate change on suitable plant distribution areas provides a valuable theoretical foundation for the effective conservation of medicinal plant diversity. Therefore, it is essential to utilize ecological models to identify potential distribution areas for medicinal plants and plant cultivation. Niche model is based on actual species distribution and environmental climate factors to predict the prediction of species through model simulations.^4^ The model employed in this study to predict the future distribution of plants is the maximum entropy (MaxEnt) model. The MaxEnt model is a model based on the principles of ecological niche principle.^5^ It can analyze and predict the potential geographical distribution pattern of species and fit the maximum entropy of the probability distribution using the data on species distribution locations and environmental variables,^6^ which is an accurate and efficient method to obtain the potential geographical distribution of species.^7^ Compared to Ecological Niche Factor Analysis (ENFA), the bioclimate analysis system (BIOCLIM), and Genetic Algorithm for Rule-set Prediction (GARP),^8^ better prediction results can be achieved even in the absence of species distribution coordinates.^9^

*Suaeda salsa* is an annual herbaceous succulent euhalophyte with high salt tolerance.^10^ The seedlings of *S. salsa* can be utilized as vegetables or as storage feed, and the leaves of the plant contain high soluble dietary fiber, and flavonoids can be extracted for anti-cancer, anti-inflammatory, and other diseases.^11^ *S. salsa* can be utilized not only in the food industry, to produce pigments, drinks, etc., but also in animal husbandry, the production of feed additives.^12^ In recent years, many scholars have concentrated on the medicinal value, heredity, mitigation of heavy metal stress, and phylogeny of *S. salsa*, but there are few reports on the response of *S. salsa* distribution pattern to climate change. In order to explore the distribution pattern of *S. salsa* in different periods, the MaxEnt model of ecological niche models (ENMs) is applied in this paper to analyze the future two periods under three greenhouse gas emission scenarios: the current period (1970–2000) and two future periods (2050s: 2041–2060 and 2090s: 2081–2100). Environmental factors of three periods were used to simulate the potential habitat area of *S. salsa*, and the change of potential habitat area, centroid transfer, and conservation status under three greenhouse gas emission models at different time periods were analyzed.

## Results

### Species distribution point data and sources of environmental factors

**Figure 1:**
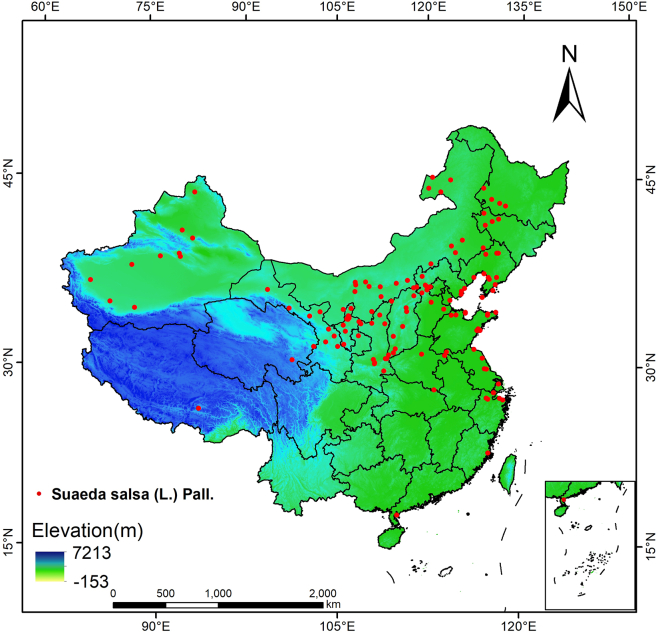
Geographical map of the distribution point of *S. salsa*

The data of *S. salsa* were primarily obtained from the Chinese Virtual Herbarium (CVH, http://www.cvh.ac.cn/) and the Global Biodiversity Information Facility (GBIF, https://www.gbif.org/).^13^ For species distribution points that lack latitude and longitude but have specific locations, the longitude and latitude coordinates of species points are determined using the Baidu Coordinate Picking System (https://api.map.baidu.com/lbsapi/getpoint/). To prevent duplicate data within the same range from affecting the accuracy of model predictions, we delete a single duplicate entries within the highest resolution based on the highest resolution of 2.5 arc minutes (approximately 5 km).^14^ This ensures that only one data point exists within every 5 km. Ultimately, the distribution data for 130 *S. salsa* were obtained (Figure 1).

A total of 37 environmental factors with a resolution of 2.5′ were obtained in this paper, including 19 climate data, 3 topographic data, and 15 soil data.^15^ Current (1970–2000) and future (2050s: 2041–2060, 2090s: 2081–2100) climate data are derived from World clim2.1 (http://www.worldclim.org/) built at Climate Model Intercomparison Project6 (CMIP6). The soil factors are obtained from the soil dataset based on the Harmonized World Soil Database (HWSD) of FAO (http://www.fao.org/faostat/en/#data), and the topographic data are derived from the Geospatial data cloud (http://www.gscloud.cn/).^16^

On the basis of the 37 environmental factors mentioned above, we considered the significance of the variables obtained through jackknife technology and quantitatively assessed the impact of these environmental factors on the geographical distribution of *S. salsa*. We employed Pearson correlation and the variance inflation factor (VIF) to evaluate the relevance and importance of the environmental factors.^17^ By using the values extracted by Spatial Analyst Tools in the arcgis10.8.1 software, spearman analysis was conducted on the spatial autocorrelation of environmental factor, and environmental factor with correlation coefficient greater than 0.7 was eliminated.^18^ According to the results of model operation, 14 environmental factors were selected to participate in the modeling (Table 1).

**Table 1:** Environmental aspects used in the modeling

Type	Variable code	Environmental factor	Unit
Climatic factor	Bio4	temperature seasonality	/
	bio5	max Temperature of Warmest Month	°C
	bio6	min Temperature of Coldest Month	°C
	Bio7	temperature annual range	°C
	Bio10	mean temperature of warmest quarter	°C
	Bio11	mean temperature of coldest quarter	°C
Soil factor	T_bs	topsoil Base SaturationBasic saturation	%
	T_caco3	topsoil Calcium Carbonate	%
	T_caso4	topsoil Gypsum	%
	T_cec_clay	topsoil CEC(clay)	cmol/kg
	T_gravel	topsoil Gravel Content	%
	T_oc	topsoil Organic Carbon	%
	T_silt	topsoil Silt Fraction	%
	T_ece	topsoil Salinity(Elco)	dS/m

T, topsoil.

The Coupled Model Intercomparison Project (CMIP) is a crucial tool for climate simulation and prediction.^19^ It involves multiple models and model centers from around the world that work together to provide data and analysis for understanding and forecasting climate change. Compared to CMIP5, CMIP6 has seen improvements in many aspects.^20^ This will help promote international cooperation and exchange in the field of climate change research.^21^ Three combined scenarios, SSP1-2.6, SSP2-4.5, and SSP5-8.5, were selected in this study. SSP1-2.6 represents the low radiative forcing scenario, whereas SSP2-4.5 represents the medium radiative forcing scenario, representing the compromise carbon emission scenario of medium social vulnerability and medium forcing.^22^ In contrast, SSP5-8.5 is a high-forcing climate scenario characterized by high population growth, high carbon emissions, and the most severe global warming trends.^23^ However, due to the loss of soil and topographic data in the future period, this study conducted a suitable area prediction on the premise that soil and topographic factors would not change in the next 70 years.^24^

### Construction of MaxEnt model and Marxan model

**Figure 2:**
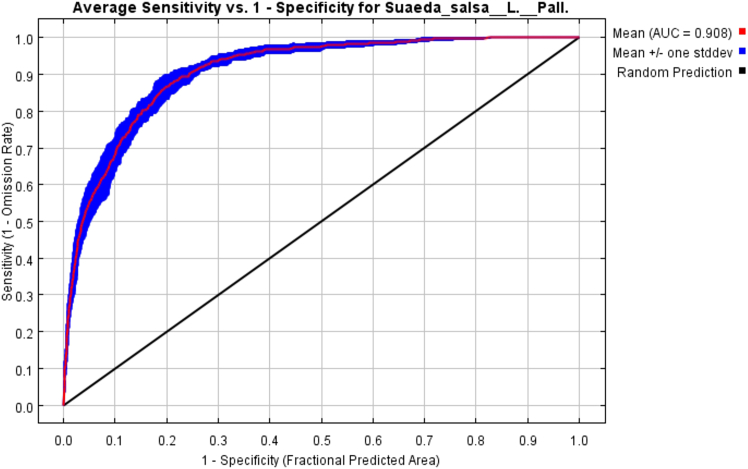
ROC response curve under the MaxEnt model

Import valid species distribution samples and filtered environmental variable data into MaxEnt 3.4.4. The parameters of the MaxEnt model are set to Set Output format to “Logistic.”^25^ Select “bootstrap” as the repeat type. Select Random seed. Set Random test percentage to 75%. Except for the abovementioned parameters, use the default parameters.^26^ The accuracy of the model is evaluated by the area under the curve (AUC) values. The AUC value in the receiver operating characteristic curve (ROC) is used to evaluate the accuracy of the model.^27^ The value of AUC ranges from 0 to 1, and the closer the value of AUC is to 1, the higher the reliability of the model. The model was reconstructed to simulate the appropriate *S. salsa* region, and the AUC value of the simulation training parameter was 0.908 (Figure 2), indicating that the prediction result was accurate.

The Marxan model draws on the basic concept of the simulated annealing algorithm at runtime to select the globally optimal solution from all possible units of solutions.^28^ This model can be used to delimit protected areas within certain cost constraints, to help construct, design, and evaluate protected areas, often used in the establishment of nature reserves.^29^ The Marxan is mainly used to develop a conservation plan based on spatial information of an area. On this basis, we have determined the location, planning units, and cost settings of the priority protection areas of the *S. salsa* in the current period. In this study, a grid of 25 km × 25 km was set as the planning unit, and then the appropriate habitat area of the target species in each planning unit was calculated by using the Zonal Statistics as Table tool in ArcGIS 10.8.1 as the protection cost of each planning unit. According to the 10%–30% threshold of the International Union for Conservation of Nature (IUCN) Terrestrial Ecosystem Conservation Plan (TECP), 30% of the upper limit of the species distribution range was chosen as the optimized conservation ratio.^30^ The conservation target was set at 30% of the total habitat area with an SPF of 100. The boundary length modifier (BLM) of the model is a correction parameter of the boundary length of the protected area.^31^ In this study, we selected a BLM value of 20,000, an SPF value of 100, and 100 model iterations to obtain the optimal solution for the planning unit. Subsequently, we imported the results into ArcGIS 10.8.1 to construct a planning map of the *S. salsa* reserve.

### Changes in spatial pattern of suitable distribution area for *S. salsa*

Load MaxEnt operation results in the ArcGIS 10.8.1 software, and use the “Spatial Analyst Tools-Reclass” tool to divide MaxEnt operation results into the following categories by natural breakpoint method: 0–0.47 is the unsuitability area, 0.47–0.55 is the low suitability area, 0.55–0.65 is the moderate suitability area, and 0.65–1 is the high suitability area.^32^ Therefore, the spatial unit of species existence probability ≥0.47 is set as the suitable region and is set as 1, and the spatial unit of species existence probability <0.47 is set as the unsuitable region. Using ArcGIS 10.8.1 software for *S. salsa* potential distribution under the condition of current and future climate data for binarization processing, calculate the *S. salsa* potential adaptation area changes in different periods, defined as four types: new suitable area, loss of suitable areas, preserve suitable areas, and uninhabitable area.^33^ In the future climate scenario, the change of the suitable area is defined as follows: 0→1 means that the suitable area is added, 1→0 means that the suitable area is reduced, 1→1 means that the suitable area is retained, and 0→0 means that the suitable area is not suitable.^34^

### Predictions of suitable areas in current and future climates of *S. salsa*

**Figure 3:**
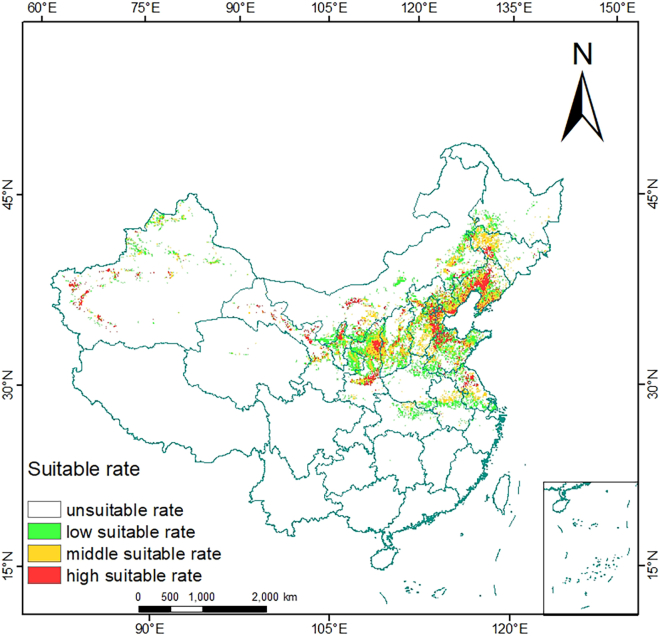
Suitable distribution of *S. salsa* in China via MaxEnt

The ArcGIS 10.8.1 software was employed to classify the current distribution area of *S. salsa* and the distribution map under the prevailing climate conditions, as illustrated in Figure 3. The species is primarily concentrated in the eastern and central regions of China, including the provinces of Jilin, Liaoning, Hebei, Beijing, Inner Mongolia, Tianjin, Shandong, Jiangsu, Anhui, Henan, Shanxi, Shaanxi, and Xinjiang. Among these, highly suitable areas are predominantly found in south-central Liaoning, eastern Hebei, northern Jiangsu, and northern Shaanxi, whereas moderately suitable areas are mainly located in western Liaoning, south-central Hebei, northeastern Shaanxi, central Shanxi, and central Anhui. As shown in Table 2, the suitable area under the current climate is 899,673 km^2^, which accounts for 9.3% of China’s total land area. Among this, the highly suitable area measures 205,011 km^2^, representing 2.1% of the total land area of China.

**Table 2:** Suitable area of *S. salsa* under different climatic scenarios (×10^4^ km^2^)

Climate change scenarios	Period	Unsuitable area	Low-grade suitable area	Moderately suitable area	Highly suitable area	Total suitable area
Current	1970–2000	870.03	36.85	32.61	20.50	89.96
SSP126-2050s	2041–2060	848.65	48.29	41.09	21.95	111.34
SSP245-2050s		859.01	6.04	74.04	20.89	100.98
SSP585-2050s		863.36	41.25	31.80	23.57	96.63
SSP126-2090s	2081–2100	868.35	39.38	30.38	21.88	91.64
SSP245-2090s		857.85	43.33	37.65	21.15	102.14
SSP585-2090s		851.39	49.53	34.86	24.19	108.60

**Figure 4:**
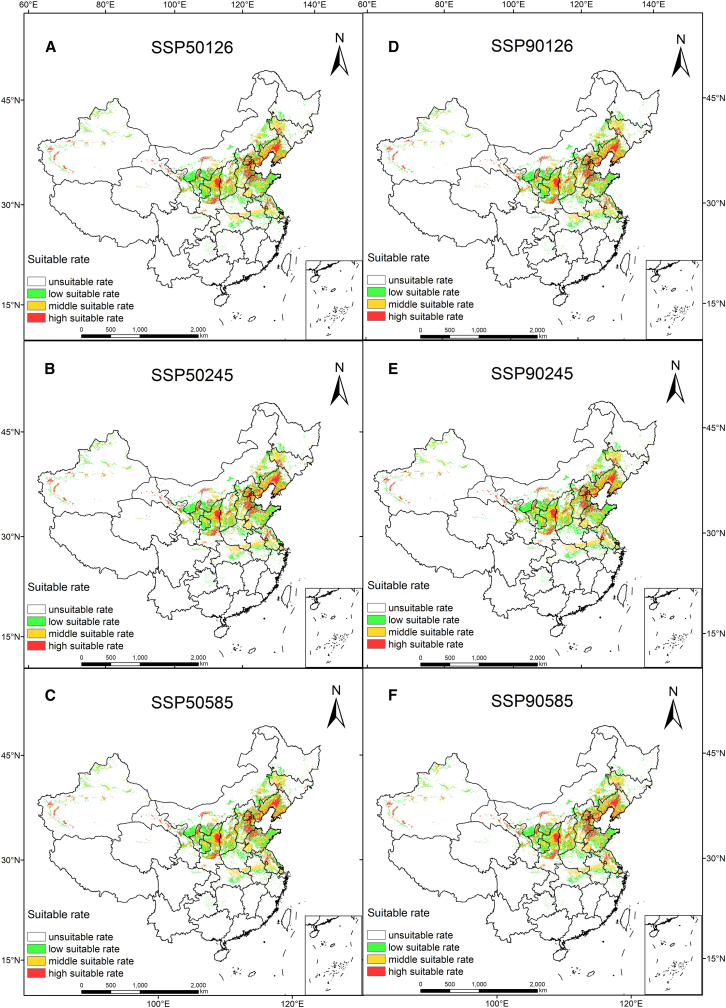
Suitable habitat distribution for *S. salsa* in China in the future under different climate change scenarios (A–C) The changes of different suitable habitats in *S. salsa* under three scenarios in the 2050s. (D–F) The changes of different suitable habitats in *S. salsa* under three scenarios in the 2090s.

The prediction results of the MaxEnt model were converted into gridded data, and the area of suitable habitat for *S. salsa* under different climate scenarios was calculated, as shown in Figure 4. Compared to the current period, the area deemed suitable for *S. salsa* in the future exhibits an overall expansion trend, with the total suitable area reaching 108.6 × 10^4^ km^2^, representing a growth rate of 20.72%. However, the changes vary over time. In the future SSP126 scenario, the total suitable area of *S. salsa* shows a trend of expanding first and then contracting over time. However, the total area still expands by 1.68 × 10^4^ km^2^, and the expanded area is mainly concentrated in North China. In the future SSP245 scenario, the total suitable area of *S. salsa* shows a slow expansion trend, with an area expansion of 12.18 × 10^4^ km^2^. In the future SSP585 scenario, the total suitable area will still show an expanding trend, and with the change of time, the expanded area will become larger and larger, with the total area expanding by 18.84 × 10^4^ km^2^, mainly concentrated in provinces such as Hebei, Shanxi, Beijing, and Shandong.

### The main environmental factors restricting the suitable area of *S. salsa* and the environmental characteristics of suitable areas

**Figure 5:**
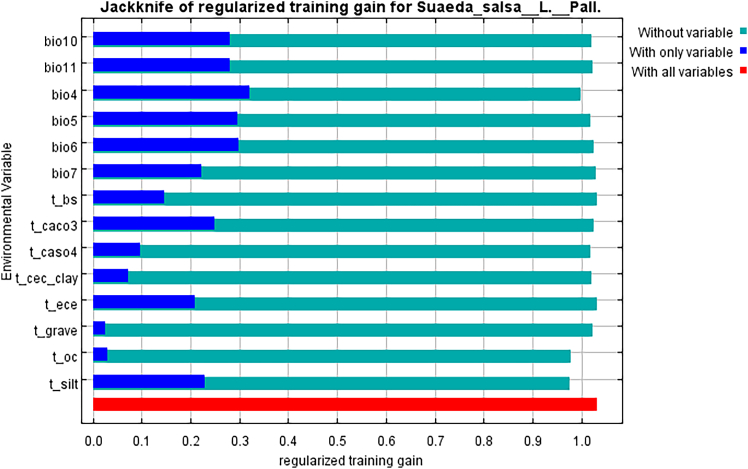
Important analysis of environmental variables based on Jackknife of regularized test gain *S. salsa*

In Figure 5, the jackknife test showed that *S. salsa* distribution was mainly affected by mean temperature of warmest quarter (Bio 10), mean temperature of coldest quarter (Bio 11), and temperature seasonality (Bio 4).

To determine important environment variables, the average contribution of each parameter was tested using a jackknife test (Table 3). The results show that the environmental variables that had the greatest influence on the growth of *S. salsa* were the temperature seasonality (22.3%), the mean temperature of warmest quarter (15.8%), the silt content in topsoil (8.7%), the exchangeable sodium salt (8.7%), the sulfate content (6.9%), the max temperature of warmest month (6.6%), the mean temperature of coldest quarter (6.2%), and temperature annual range (5.6%). The total contribution rate of eight environmental factors is 80.8%, among which the seasonal change of temperature (21.3%) is the most significant variable affecting the distribution. From the perspective of important value and contribution, temperature factor has the most significant influence, followed by soil and slope variability factor. Precipitation factors and elevation had little effect on the distribution of *S. salsa.*

**Table 3:** Contribution rates and importance values of environmental factors

environmental factor	Contribution rate (%)	Importance rate (%)
Temperature seasonality	22.3	33.4
Mean temperature of warmest quarter	15.8	16
Silt content in topsoil	8.7	5.9
Exchangeable sodium salt	8.7	1.1
Sulfate content	6.9	3.9
Max temperature of warmest month	6.6	6.8
Mean temperature of coldest quarter	6.2	7.6
Temperature annual range	5.6	2.6
Basic saturation	4.6	1.5
Carbonate or lime content	4.6	1.2
Organic carbon content in topsoil	4.2	5.9
Min temperature of coldest month	4.2	11.3
Gravel volume in topsoil	0.9	1.7
Cation exchange capacity of cohesive soil	0.8	1.2

**Figure 6:**
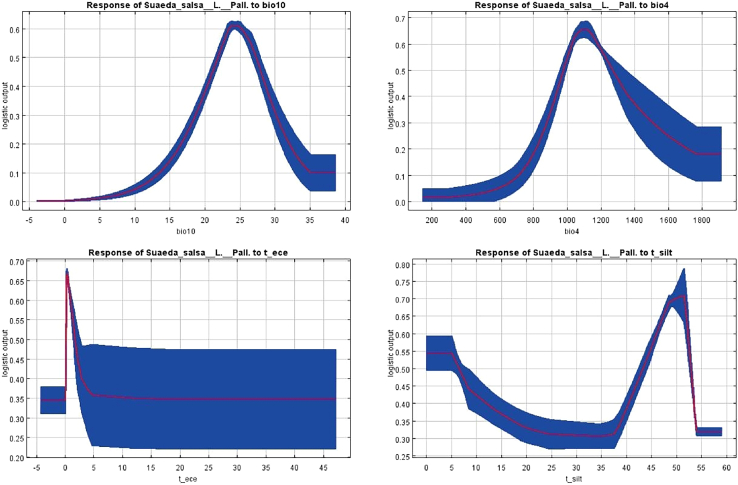
Single-factor response curve of the current climate

In order to further clarify the climate characteristics of the potential suitable area of the *S. salsa* based on current climate conditions, the response curves of four environmental factors that have significant influence on the geographical distribution of the *S. salsa* were further studied, as shown in Figure 6. It is generally believed that when the survival rate is greater than 0.5, the corresponding environmental factors are suitable for plant growth. We found that the temperature curves for the seasonal change in temperature (bio4) and the average temperature in the warmest quarter (bio10) show very similar trends. In the warmest season, when the average temperature is kept in the range of 22°C–28°C, the survival rate is the best, and when the average temperature is close to 25°C, the survival rate is the highest. When the seasonal temperature variation range is 1,000–1,200, the survival rate of *S. salsa* is the highest, and when the seasonal temperature variation range is 200–800 or greater than 1,600, the survival rate of *S. salsa* is lower. In soil factors, the topsoil silt fraction (t_silt) and topsoil salinity (t_ece) in topsoil are different. In the topsoil silt fraction, *S. salsa* can survive well in the range of 0%–5%. When it is 5%–35%, the survival rate of *S. salsa* gradually decreases to the lowest point. In the topsoil salinity, the curve remains unchanged at −5 to 0 dS/m, and the survival rate of *S. salsa* is the lowest and remains unchanged. At 0–1 dS/m, the curve rose rapidly to the highest point, and the survival rate of the *S. salsa* was the highest. At 1–5 dS/m, the curve dropped sharply, and so did the survival rate.

### Dynamic changes of suitable habitat of *S. salsa* under different climate scenario combinations

**Figure 7:**
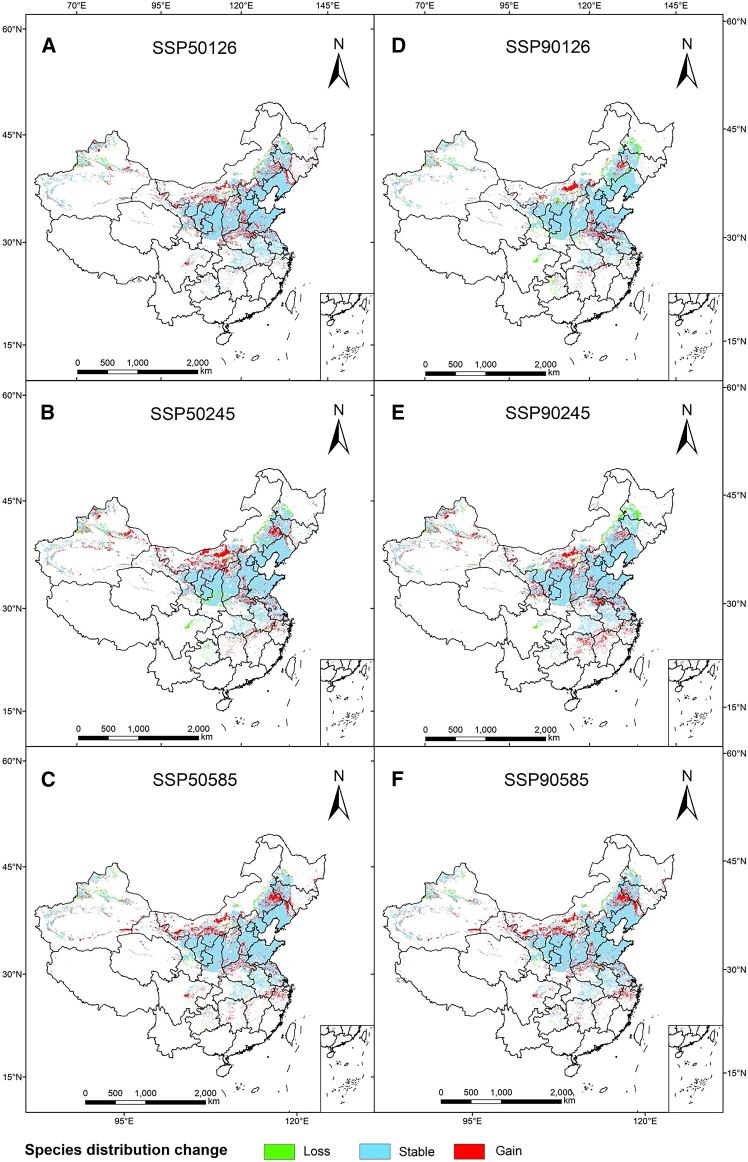
Dynamic change map of the predicted potentially suitable area of *S. salsa* (A–C) The changes of the total suitable area of *S. salsa* under different scenarios in the 2050s. (D–F) The changes of the total suitable area of *S. salsa* under different scenarios in the 2090s.

A comparative analysis of the spatial pattern of the suitable area for *S. salsa* under six different climate scenarios in the future shows that (Figure 7) under different climate scenarios, the suitable area for *S. salsa* will experience different degrees of reduction in different periods in the future (Table 4). Nevertheless, the vast majority of current suitable areas will remain suitable. Under the three future climate scenarios, the range of suitable habitat loss was 10.93%–21.13%, the habitat loss area was 4.7–13.96 × 10^4^ km^2^, and the land loss was mainly concentrated in Northeast, North, and central China, including Heilongjiang, Henan, and Hubei provinces (Figure 7). The area of suitable area increased by 18.09–34.98 × 10^4^ km^2^, with a growth rate of 2.49%–16.95%. The expansion of suitable habitat is mainly concentrated in the northern regions of Xinjiang, Henan, and Hebei. The regional change rate ranges from 2.84% to 8.43%, with the lowest change rate under SSP126-2050 climate scenario and the highest change rate under SSP126-2090 climate scenario. In the SSP126-2090 climate scenario, the maximum loss of the appropriate area is 13.96 × 10^4^ km^2^, and the minimum increase is 18.09 × 10^4^ km^2^, indicating that the climate scenario is more sensitive to climate change. Under the SSP126-2050 climate scenario, the loss of suitable salt-soil canopy area also reaches the minimum. The comparative analysis of potential habitat spatial pattern changes under different climate change scenarios in the future shows that the spatial area change rate of potential habitat area of peony in the 2090s is greater than that in the 2050s, indicating that the spatial pattern change of potential habitat in the 2090s is more significant.

**Table 4:** Changes in the distribution area of *S. salsa* under different periods and scenarios

Period	Climate scenario	Habitat area (×10^4^ km^2^)	Loss (×10^4^ km^2^)	Stable (×10^4^ km^2^)	Gain (×10^4^ km^2^)	Species range change (%)	Percentage loss (%)	Percentage gain (%)
Current		165.55						
2041–2060	SSP126	192.99	4.70	160.85	32.14	−2.84	19.41	16.57
	SSP245	190.77	9.77	155.78	34.98	−5.90	21.13	15.23
	SSP585	193.63	6.24	159.31	34.31	−3.77	20.72	16.95
2081–2100	SSP126	169.68	13.96	151.58	18.09	−8.43	10.93	2.49
	SSP245	189.59	8.86	156.69	32.90	−5.35	19.87	14.51
	SSP585	193.63	6.24	159.31	34.31	−3.77	20.72	16.95

### Changes in the centroid of potentially suitable areas

**Figure 8:**
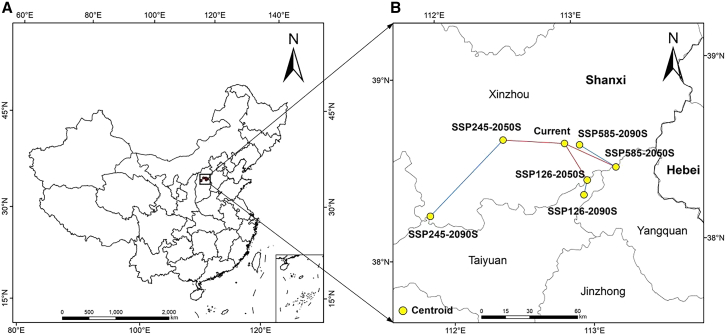
Centroids of the predicted potentially suitable area of *S. salsa* (A) The changes of *S. salsa* centroids in China in different eras. (B) The changes of *S. salsa* centroids in Shanxi Province in different eras.

In this study, the potential habitat area of *S. salsa* was defined as the geometric center point to simulate the change of centroid migration under different climate scenarios. As can be seen from Figure 8, the position of the center of mass in the future period is transferred in different directions compared with the position of the current center of mass. In the future SSP1-2.6 scenario, the centroid position in the 2050s will migrate to the southeast direction compared with the current centroid position, and the migration distance is 26.60 km (Table 5). The position of the center of mass in the 2090s has shifted 9.31 km to the southwest compared to the position of the center of mass in the 2050s. In the future SSP2-4.5 scenario, the position of the centroid in the 2050s migrates to the northwest direction compared to the current centroid, and the migration distance is 63.45 km. Compared with that of the 2050s, the centroid of the 2090s migrates to the southwest, and the migration distance is 50.02 km. In the future SSP5-8.5 scenario, the position of the centroid in the 2050s migrates to the southeast direction compared to the current centroid, and the migration distance is 35.82 km. Compared with the 2050s, the center of mass in the 2090s migrates to the northwest, and the migration distance is 26.87 km.

**Table 5:** Distribution of centroid longitude and latitude of *S. salsa* in different periods

Period	Current	2050s			2090s		
		SSP126	SSP245	SSP585	SSP126	SSP245	SSP585
Longitude	112.887	113.029	112.441	113.246	112.998	111.874	112.995
Latitude	38.582	38.371	38.627	38.4300	38.291	38.236	38.568

## Discussion

### Potential geographical distribution changes of *S. salsa* and resource conservation

The suitable habitat for plants on Earth is greatly limited by climatic conditions.^35^ By comparing the distribution and changes in suitable habitat for different types of *S. salsa* in both current and future periods, and by observing the movement of their geographical centroid, it is evident that the total area of suitable habitat for *S. salsa* has increased under various climate scenarios compared to the present. This suggests that the distribution and changes in suitable habitat for *S. salsa* under higher concentration emission scenarios may be more conducive to climate conditions. The expansion of the most suitable habitat was primarily concentrated in the northern regions of Xinjiang, Shanxi, and Henan, which included an overall shift of the centroid (Figure 8). In the 2050s, the area of the suitable area under the three climate scenarios did not change much, but in the 2090s, the area of the suitable area under the three climate scenarios increased, especially the area of the low-degree suitable area showed an increasing trend, indicating that the area of the distribution of the *S. salsa* is getting larger and larger. Over time, the distribution area of the *S. salsa* has increased rapidly, so it can be inferred that the situation of global warming is gradually intensified. The spatial pattern of the appropriate area responds to climate change in general, that is, with the intensification of climate warming, the spatial migration range of the overall spatial location of the appropriate area increases. The size of niche overlap reflects the similarity of resource use by plants.^36^ The large niche overlap suggests that they have similar ecological needs, resource use, and biological characteristics under certain circumstances. The reduction of co-available resources in the future and current periods indicates that the geographical distribution area of *S. salsa* in China has changed under the influence of global climate change, which is consistent with the overall migration of the mass center in the suitable area predicted by the MaxEnt model. Compared with the current period, the niche width of the *S. salsa* increased under the future climate scenario, indicating that its suitable area increased under the influence of global change, which is consistent with the predicted results of the model, proving the accuracy of the model.^37^

### Important environmental factors affecting the suitable area of *S. salsa*

By synthesizing Percent Contribution (PC) values, Permutation Importance (PI) values and jackknife values,^38^ the results indicated that temperature seasonality, the mean temperature of the warmest quarter, the silt content in topsoil, and exchangeable sodium salt were the most important environmental variables affecting the potential suitable habitat of the *S. salsa*. The results show that the most suitable habitat of *S. salsa* in China is mainly distributed in the subhumid monsoon climate zone with temperature of 15°C–25°C and about 25°–35° north latitude. Its growth characteristics are consistent with the main environmental factors that affect the growth of *S. salsa*. Under current climate conditions, temperature is the main environmental factor affecting climate distribution, followed by soil factor, and precipitation factor has the least influence. According to the response curve of each factor, it is determined that the highest temperature in the suitable area of the *S. salsa* can reach 35°C and the lowest temperature can reach −4°C, which is consistent with the temperature in the distribution area of the *S. salsa* in China. Although the hydrothermal conditions play an important role in the potential geographical distribution pattern of the *S. salsa* in China, the constraints of topographic and soil factors cannot be ignored. In this study, the high suitability area was mainly concentrated in northern China, which coincided with the optimal growth area of the *S. salsa*, which verified the accuracy of the results. Hydrothermal conditions are important variables that limit the geographical distribution of plants, and terrestrial plants are more sensitive to temperature.^39^ The results of MaxEnt model show that temperature is the most important environmental factor limiting the potential geographical distribution of *S. salsa*, accounting for 72.9%. Temperature mainly affects the activity of enzymes related to plant photosynthesis.^40^ These enzymes play a catalytic role in photosynthesis, and their activity varies with temperature. In a certain range, with the increase of temperature, the activity of enzyme is enhanced and the rate of photosynthesis is accelerated. However, when the temperature is too high, the activity of the enzyme will be inhibited, and even lead to enzyme denaturation and inactivation, thus reducing the rate of photosynthesis. Secondly, the soil factor is also an important factor affecting the survival of the salt soil. Topsoil silt content has an important effect on plant growth and soil quality. Appropriate amount of sediment can increase the soil permeability and water retention, which is conducive to the growth and development of plant roots. However, if the sediment content is too high, it may cause the soil structure to become compact, affecting the soil’s air permeability and water retention, which will adversely affect the growth of plant. In addition, high sediment content may also affect the nutrient content and pH of the soil, and the development of plant communities largely depends on the availability of soil nutrients.^41^ As a salt-tolerant plant, the content of salt in soil also has an important effect on the growth of salt-tolerant plant.^10^ At the same time, the inorganic elements such as Mn, Fe, Zn, and Cu in the soil play a vital role in the growth of the *S. salsa*. Therefore, the influence of temperature, soil, and precipitation on the potential distribution pattern of *S. salsa* cannot be ignored.

### Priority protected area of *S. salsa*

**Figure 9:**
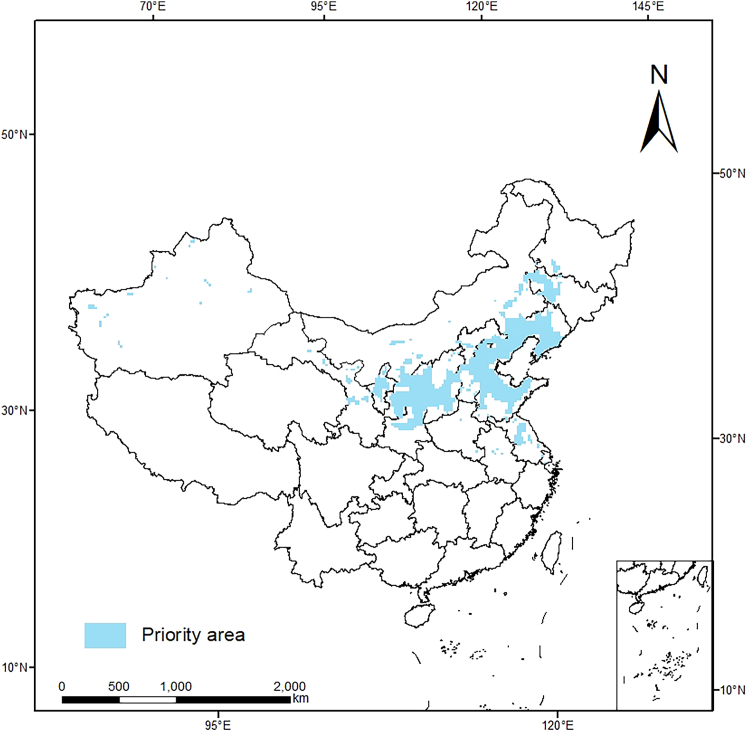
Priority protected areas of *S. salsa* in China predicted by the Marxan model

From the point of view of nature reserve, the systematic protection of hemp was discussed.^42^ The results showed that the priority protected areas of *S. salsa* were concentrated in Hebei, central and southern Liaoning, northern Shandong, Shanxi, and eastern Shaanxi. Most of these areas are northern regions, with less precipitation, large monthly temperature differences, good light conditions, and some areas are more arid, which are consistent with the growth of most medicinal plants, are very suitable for the growth of Chinese herbs. The establishment of nature reserves is more conducive to plant protection with less human interference. At the same time, the priority protected areas have certain suitable conditions for the growth of *S. salsa*, which is of guiding significance for industrial development. In the future development, it is also possible to take the development of *S. salsa* planting base as the primary task of local promotion economic development. The large niche overlap indicates that they have similar ecological needs, resource use, and biological characteristics under certain circumstances. The decrease in the resources available for common use in the future and current periods indicates that the geographical distribution area of *S. salsa* in China has changed under the influence of global climate change, which is consistent with the overall migration of the centroid of the suitable area predicted by the MaxEnt model (Figure 9). The species all have similar morphological and physiological characteristics, and their geographical distribution also has some similarities in coping with climate change. Therefore, the model has certain guiding significance for predicting the distribution area of other species under future climate scenarios.

### Limitations of the study

This study has several inherent constraints. First, the model assumes that soil and topographic conditions remain constant over future periods, which may not reflect real-world changes. Second, the occurrence data used for model training may include sampling bias due to uneven field collection. Third, only 14 environmental variables were selected based on statistical thresholds, potentially excluding factors that could influence species distribution. Additionally, the Marxan model does not incorporate socioeconomic constraints or land use conflicts, which may affect the feasibility of proposed conservation zones. Future studies should consider integrating dynamic environmental datasets, land use projections, and broader ecological interactions to enhance prediction accuracy and practical applicability.

### Conclusion

As a medicinal and saline-resistant plant, *S. salsa* is mainly distributed in central and northeastern China, including the provinces of Liaoning, Shanxi, Shaanxi, Hebei, Shandong, Inner Mongolia, Xinjiang, and other provinces. Temperature and soil conditions are the main environmental factors that affected the distribution of *S. salsa* plants. With the intensification of global warming, in the future climate conditions, the area suitable for growth will increase, but the suitable area in a few areas will shrink and migrate to the eastern region as a whole. The priority protected areas are mainly distributed in Hebei, central and southern Liaoning, northern Shandong, Shanxi, and eastern Shaanxi, which are basically consistent with the highly suitable protected areas predicted by MaxEnt model. Therefore, the planning of the nature reserves must take into account the impact of future climate change and move moderately to the northeast based on the current forecast area. In this study, when predicting future climate distribution in this region, there is no change in the data except the climate data, which may lead to some bias. Although the model may have some inadequacies, it still has important guidance and reference significance for the protection of *S. salsa*.

## Resource availability

### Lead contact

Further information and requests for resources should be directed to and will be fulfilled by the lead contact, Dr. Fenguo Zhang (zhangfenguo@sxnu.edu.cn).

### Materials availability

This study did not generate new unique materials.

### Data and code availability

• All data used in this study are publicly available. Occurrence data of *Suaeda salsa* were obtained from the Chinese Virtual Herbarium (http://www.cvh.ac.cn/) and the Global Biodiversity Information Facility (https://www.gbif.org/). Climate data were sourced from WorldClim 2.1 (https://www.worldclim.org/), and soil/topographic data were obtained from the Harmonized World Soil Database and Geospatial Data Cloud. This paper does not report original code.
• Any additional information required to reanalyze the data reported in this paper is available from the lead contact upon request.

## Acknowledgments

This work was supported by the National Natural Science Foundation of China (Grant No. 41801027 and No. 31700434), the Fundamental Research Program of Shanxi Province (Grant No. 202203021211250 and No. 202203021211252), the Fund Program for the Scientific Activities of Selected Returned Overseas Professionals in Shanxi Province (Grant No. 20230025 and No. 20230027), the Research Project Supported by Shanxi Scholarship Council of China (Grant No. 2024-089 and No. 2023-110), and the Science and Technology Innovation Project of Colleges and Universities in Shanxi Province (2021L274).

## Author contributions

Y.W., conceptualization, methodology, software, formal analysis, investigation, and writing—original draft. Z.L., writing—review and editing, supervision, methodology, and resources. K.W., resources. J.P., software and writing. Y.M., software and writing. G.Z., software, methodology, and formal analysis. F.Z., funding acquisition, supervision, writing, review, and editing.

## Declaration of interests

The authors declare that the research was conducted in the absence of any commercial or financial relationships that could be construed as potential conflicts of interest.

## STAR★Methods

### Key resources table

**Table:** 

REAGENT or RESOURCE	SOURCE	IDENTIFIER
**Biological samples**		

Suaeda salsa occurrence records	Chinese Virtual Herbarium Database (CVH)	http://www.cvh.ac.cn
Suaeda salsa occurrence records	Global Biodiversity Information Facility Database (GBIF)	https://www.gbif.org

**Software and algorithms**		

MaxEnt(version 3.4.4)	American Museum of Natural History	https://biodiversityinformatics.amnh.org/open_source/maxent/
ArcGIS (version 10.8.1)	Esri	https://desktop.arcgis.com/es/arcmap/latest/get-started/installation-guide/installing-on-your-computer.htm
Marxan	University of Queensland	https://marxansolutions.org/
R (version 4.3–1)	R Core Team	https://cran.r-project.org/bin/windows/base/old/4.3.1/

**Data Resources**		

Bioclimatic data (current and future)	WorldClim Database v2.1	https://www.worldclim.org/
Soil and topographic data	Harmonized World Soil Database & Geospatial Data Cloud Database	http://www.fao.org/soils-portal, http://www.gscloud.cn

### Experimental model and study participant details

This study did not involve animal or human participants. The experimental model used was the halophytic plant Suaeda salsa (L.) Pall., a widely distributed annual herbaceous species in saline habitats of China. Occurrence records of S. salsa were obtained from publicly available biodiversity databases, including the Chinese Virtual Herbarium (CVH, http://www.cvh.ac.cn/) and the Global Biodiversity Information Facility (GBIF, https://www.gbif.org/). Each occurrence record was carefully validated to ensure accurate geographic coordinates. No direct handling, cultivation, or treatment of live specimens was performed in this study, and all data used were collected from open-access repositories. Therefore, institutional permissions and ethical oversight were not required.

### Method details

#### Species selection and occurrence data

*Suaeda salsa* (L.) Pall(*S. salsa*)*.,Amaranthaceae (formerly Chenopodiaceae),.genus*. *S. salsa* is an annual herbaceous plant, typically 20–50 cm in height, with erect or sprawling stems that branch frequently, displaying green to purplish-red coloration.^43^ Its fleshy, linear or cylindrical leaves are smooth and range from gray-green to reddish-purple, an adaptation for water storage in saline environments. The small, inconspicuous flowers grow in clusters at leaf axils, blooming from July to September. The seeds are nearly round, dark brown, and shed easily upon maturity^44^.*S. salsa* is a typical halophyte that can grow in coastal tidal flats and saline-alkali wastelands with soil salinity levels of 1%–3%, even regulating internal salt concentrations through salt-secretion mechanisms.^45^It is widely distributed in coastal regions of China (e.g., North China, Northeast China) and other saline-alkali areas.As a pioneer species in saline-alkali soil remediation, it promotes soil improvement by accumulating organic matter. Its root system helps stabilize saline soils, reducing erosion, while its salt absorption lowers soil salinity.^46^ Additionally, its tender stems and leaves are rich in amino acids and minerals, making them edible as a wild vegetable (locally known as “Yanhao”). In traditional medicine, it is used to clear heat and relieve summer heat, giving it high medicinal value.^47^

The occurrence records of *S. salsa* were obtained from the **Chinese Virtual Herbarium (CVH,** http://www.cvh.ac.cn/**)** and the **Global Biodiversity Information Facility (GBIF,** https://www.gbif.org/**)**.^48^ However, the raw dataset exhibited significant spatial duplication, primarily due to sampling bias in field collections.To mitigate spatial sampling bias while retaining maximum ecological information, we applied **spatial thinning** using the **SpThin package in R**,^49^ based on the highest resolution (2.5arc-minutes,∼5km) of WorldClim (www.worldclim.org) environmental layers.^50^ This method ensured that only one occurrence record was retained per 5 × 5 km grid cell, effectively reducing autocorrelation while preserving biogeographical representativeness.After processing, a refined dataset of **130 spatially independent occurrence records** was obtained for subsequent species distribution modeling.

#### Selection of environmental variables

A total of 37 environmental factors with a resolution of 2.5′ were obtained in this paper, including 19 climate data, 3 topographic data and 15 soil data^51^ Current (1970–2000) and future (2050s: 2041–2060, 2090s: 2081–2100) climate data are derived from World clim2.1 (http://www.worldclim.org/) built at Climate Model Intercomparison Project6(CMIP6).^52^The Coupled Model Intercomparison Project (CMIP) serves as a critical tool for climate simulation and prediction.^53^ This international framework involves multiple climate models and modeling centers worldwide, collectively generating data and analyses to enhance understanding and projection of climate change. Compared to its predecessor CMIP5, CMIP6 demonstrates significant improvements and advancements across multiple dimensions.^54^For this study, we selected three representative combined scenarios:SSP1-2.6: Represents a low radiative forcing pathway, aligning with sustainable development goals and rapid decarbonization.SSP2-4.5: An intermediate radiative forcing scenario, reflecting a balanced trajectory of moderate societal vulnerability and mitigated emissions.SSP5-8.5: A high-end forcing scenario characterized by high population growth, intensive fossil-fuel dependency and the most severe projected global warming trends.^55^This selection enables comprehensive assessment of climate impacts across plausible future trajectories, from stringent mitigation to unconstrained development pathways. The SSP-RCP framework provides consistent integration of socioeconomic factors with radiative forcing levels, offering more policy-relevant projections than CMIP5’s standalone RCP approach.^56^

We conducted environmental variable selection through a rigorous statistical screening process using ArcGIS 10.8.1 and R programming platforms. Initially, we employed the Reclassify tool in ArcGIS 10.8.1 to extract numerical values for spatial autocorrelation analysis.^57^ Using Spearman’s rank correlation analysis,^58^ we eliminated environmental variables with correlation coefficients exceeding 0.7 to reduce multicollinearity effects.Building upon the initial set of 37 environmental factors, we incorporated variable importance assessments through jackknife tests to quantitatively evaluate their influence on the geographic distribution of *S. salsa*.^59^ The screening process involved two complementary statistical approaches: (1) Pearson correlation analysis and (2) variance inflation factor (VIF) tests. Through Spearman correlation analysis and VIF multicollinearity analysis of the point-interpolated data in R,^60^ we performed preliminary screening to retain only variables meeting the following criteria: correlation coefficients <0.7 and VIF values < 5. During this exclusion process, we strategically preserved factors known to be ecologically significant for plant distribution to enhance model accuracy.

#### Modeling with MaxEnt

The Maximum Entropy (MaxEnt) model holds significant advantages over other models in predicting future species distributions.^37^ It demonstrates robust performance with small sample sizes, capable of building reliable models even with only 10–20 species occurrence records. The model operates on the principle of maximum entropy, effectively filling gaps in data while avoiding overfitting.MaxEnt excels at capturing complex relationships among environmental variables, automatically detecting threshold effects and interactions (e.g., temperature-precipitation synergies). Compared to the GARP model,^8^ MaxEnt better handles incomplete environmental data by using pseudo-absence points (background points) instead of requiring true absence records. Unlike the BIOCLIM model, MaxEnt employs regularization to reduce unrealistic predictions under extreme climate scenarios, showing greater stability with high-variability CMIP6 climate data. Under the high-emission SSP5-8.5 scenario, MaxEnt exhibits lower collapse risk in predictions compared to Random Forest.

We import the censored species distribution points and environmental variable data into MaxEnt 3.4.4. The parameters of the MaxEnt model are set as follows: Select the Output format as “Logistic”, select “bootstrap” as the repeat type, check “Random seed”, set Random test percentage to 75%, Replicates to 10000, in addition to the above parameters,^25^ The rest use default parameters. The accuracy of the model is evaluated by the value of AUC. The AUC value in the Receiver operating characteristic curve (ROC) is used to evaluate the accuracy of the model.^27^ The AUC value ranges from 0 to 1, and the closer the AUC value is to 1, the higher the credibility of the model.

#### Conservation planning with Marxan

The Marxan model incorporates the fundamental concepts of **simulated annealing algorithms** to identify globally optimal solutions from all potential planning unit combinations.^61^ It is designed to delineate protected areas under specified cost constraints, aiding in the systematic **design, construction, and evaluation** of conservation networks, particularly for establishing nature reserves.The use of Marxan is mainly to formulate protection plans based on the spatial information of a certain area. Based on this, we have determined the location of the priority protection areas for *S. salsa* in the current period, as well as the planning units and cost Settings. In this study, a 25 km × 25 km grid was set up as the planning unit, and then the zone Statistics as Table tool in ArcGIS 10.8.1 was used to calculate the suitable habitat area of the target species within each planning unit as the conservation cost of each planning unit.^62^ According to the threshold of 10%–30% of the IUCN Terrestrial Ecosystem Conservation Program (TECP),^30^ 30% of the upper limit of the species distribution range is selected as the optimal conservation ratio. The protection target is set at 30% of the total habitat area with an SPF value of 100. The boundary length corrector (BLM) of the model is the correction parameter of the boundary length of the protected area. By modifying the BLM,^31^ we can analyze the relationship between the cost and the total length and total area of the boundary, thereby finding a balance point and obtaining a more reasonable spatial distribution pattern of the protected area through calculation. The final model used 20,000 model boundary modifiers. Iterate the model 100 times to obtain the optimal solution of the planning unit.

#### Quantification and statistical analysis

Model performance was assessed using the Area Under the Curve (AUC) of the Receiver Operating Characteristic (ROC). An AUC value above 0.9 was considered highly accurate. Spatial changes in habitat were quantified using ArcGIS 10.8.1. Reclassification and binarization were performed to analyze habitat dynamics under different climate scenarios (SSP1-2.6, SSP2-4.5, SSP5-8.5) for the 2050s and 2090s.

### Additional resources

This study did not utilize or generate additional tools, software, or databases beyond those listed above.
